# Alterations of polyunsaturated fatty acid metabolism in ovarian tissues of polycystic ovary syndrome rats

**DOI:** 10.1111/jcmm.13614

**Published:** 2018-03-30

**Authors:** Rong Huang, Xinli Xue, Shengxian Li, Yuying Wang, Yun Sun, Wei Liu, Huiyong Yin, Tao Tao

**Affiliations:** ^1^ Department of Endocrinology and Metabolism RenJi Hospital School of Medicine Shanghai JiaoTong University Shanghai China; ^2^ Key Laboratory of Food Safety Research Institute for Nutritional Sciences Shanghai Institutes for Biological Sciences Chinese Academy of Sciences Shanghai China; ^3^ Shanghai Key Laboratory for Assisted Reproduction and Reproductive Genetics Center for Reproductive Medicine RenJi Hospital School of Medicine Shanghai JiaoTong University Shanghai China; ^4^ University of the Chinese Academy of Sciences Shanghai China; ^5^ Key Laboratory of Food Safety Risk Assessment Ministry of Health Beijing China; ^6^ Mass Spectrometry Research Center Institute for Nutritional Sciences Shanghai Institutes for Biological Sciences Chinese Academy of Sciences Shanghai China; ^7^ School of Life Science and Technology ShanghaiTech University Shanghai China

**Keywords:** polycystic ovary syndrome, polyunsaturated fatty acids, cyclooxygenase, cytosolic phospholipase A2, ovarian function

## Abstract

The metabolism of polyunsaturated fatty acids (PUFAs) remains poorly characterized in ovarian tissues of patients with polycystic ovary syndrome (PCOS). This study aimed to explore alterations in the levels of PUFAs and their metabolites in serum and ovarian tissues in a PCOS rat model treated with a high‐fat diet and andronate. Levels of PUFAs and their metabolites were measured using gas/liquid chromatography‐mass spectrometry after the establishment of a PCOS rat model. Only 3 kinds of PUFAs [linoleic acid, arachidonic acid (AA) and docosahexaenoic acid] were detected in both the circulation and ovarian tissues of the rats, and their concentrations were lower in ovarian tissues than in serum. Moreover, significant differences in the ovarian levels of AA were observed between control, high‐fat diet‐fed and PCOS rats. The levels of prostaglandins, AA metabolites via the cyclooxygenase (COX) pathway, in ovarian tissues of the PCOS group were significantly increased compared to those in the controls. Further studies on the mechanism underlying this phenomenon showed a correlation between decreased expression of phosphorylated cytosolic phospholipase A2 (p‐cPLA2) and increased mRNA and protein expression of COX2, potentially leading to a deeper understanding of altered AA and prostaglandin levels in ovarian tissues of PCOS rats.

## 1 INTRODUCTION

Polycystic ovary syndrome (PCOS) is a common ovulatory disorder among women of childbearing age, with 50% of PCOS patients also being accompanied by infertility.^1^ PCOS is often associated with obesity, insulin resistance, lipid metabolic disorders and impaired glucose tolerance.^2^ In the in vitro fertilization cycle, associations between elevations in total follicular free fatty acids (FFAs) and poor cumulus‐oocyte complex quality have been found, suggesting that excess FFAs influence ovarian follicular function.^3^ Furthermore, obese women with PCOS undergoing controlled ovarian hyperstimulation have elevated oleic and stearic acid concentrations, which are related to adverse pregnancy outcome.^4^ Polyunsaturated fatty acids (PUFAs), particularly those of the n−3 and n−6 families, are perhaps the most potent fatty acid regulators of metabolic function and are implicated in a diverse range of processes in vivo.^5^ The prevalence of obesity in women with PCOS is approximately 50%‐80%,^6^ and n−3 PUFA supplementation in women with PCOS reduces the plasma bioavailable testosterone, with the greatest reductions being observed in subjects who exhibit greater reductions in plasma n−6:n−3 PUFA ratios.^7^ Furthermore, recent studies have indicated the fatty acid composition of oocytes and their environmental influence on developmental competence and pregnancy outcome.^4^, ^8^, ^9^

Eicosanoids, including prostaglandins (PGs) and leukotrienes, which are cyclooxygenase (COX)‐generated metabolites of arachidonic acid (AA),^10^ are known to modulate different ovarian functions and luteolysis.^11^, ^12^ Specifically, PGs are biologically active lipid mediators involved in chronic inflammation and regulation of many reproductive events, such as ovulation, corpus luteum regression, implantation and pregnancy establishment.^13^ Alterations in dietary PUFAs can change PUFA contents in the cell membrane and PG synthesis, thus affecting fertility.^14^ n‐3 PUFAs may be effective in improving hyperandrogenism and insulin resistance in patients with PCOS.^15^ Elevated concentrations of AA and linoleic acid (LA) in follicular fluid at the time of oocyte retrieval significantly decrease the ability of oocytes to form pronuclei after intracytoplasmic sperm injection, but levels of AA and LA are not associated with subsequent embryo quality or pregnancy rate.^16^

As acquisition of patient ovarian tissues is difficult, alterations in ovarian hormonal and metabolic disorders remain poorly defined. Lipidomics, a branch of metabolomics, can systematically investigate a broad range of lipids in one biological system, and research on ovarian PUFAs and their derivates in a PCOS rat model by lipidomics might contribute to a deeper understanding of hormonal and metabolic disorders and their mechanisms.

## 2 MATERIALS AND METHODS

### 2.1 Research subjects

Animal experiments were performed according to protocols approved by the Animal Care Committee of Shanghai Jiaotong University School of Medicine. Twenty‐three 21‐day‐old female Sprague Dawley rats (50‐60 g, Shanghai Laboratory Animal Center of Chinese Academy of Science, Shanghai, China) were separately housed in individually ventilated cages (IVC) in a temperature‐ and humidity‐controlled environment. The animals were randomly divided into 3 groups: control (CON, n = 8), high‐fat diet‐fed (HF, n = 7), and high‐fat diet‐fed and andronate‐treated (PCOS, n = 8). The models were established as previously described.^17^ Rats in the PCOS group were fed a high‐fat diet and subcutaneously injected with andronate (Sigma‐Aldrich) daily at a dose of 1 mg/100 g body weight in 0.2 mL of oil as a vehicle for 8 weeks, whereas rats of the control group were fed a normal diet and injected with only the vehicle.

### 2.2 Lipidomics

Solvents for sample preparation and mass spectrometry (MS) analysis, including methanol (MeOH), chloroform (CHCl3) and water, were purchased from Burdick and Jackson (MI, USA). Other HPLC‐quality solvents, including methanol, water, 2‐propanol, hexane and acetonitrile, were purchased from either Fisher Chemical (Phillipsburg, NJ, USA) or EM Science (Gibbstown, NJ, USA). Standards and deuterated standards for AA metabolites, including PGD2, PGE2, PGF2α, PGI2, thromboxane B2 (TXB2), hydroxyeicosatetraenoic acids (HETEs), hydroxyoctadecadienoic acids (HODEs) and epoxyeicosatrienoic acids (EETs), were purchased from Cayman Chemicals (Ann Arbor, MI, USA) and used without further purification. Fatty acids of the highest purity (>99%) were purchased from NuChek Prep (Elysian, MN, USA). All other chemical reagents were purchased from Sigma‐Aldrich (St. Louis, MO).

Fatty acids were analysed as fatty acid methyl esters (FAMEs) according to published protocols.^18^ Briefly, FAMEs were separated on an SP‐2560 capillary column (100 m * 0.25 mm i.d. * 0.2 μm film; Supelco, Bellefonte, PA, USA) and analysed by gas chromatography‐mass spectrophotometry (GC‐MS, SHIMADZU, QP 2010 Ultra) in the positive‐ion mode of electron impact.

AA metabolites were analysed using a previously reported method.^19^, ^20^ Briefly, ovarian tissues were dissected, and approximately 10 mg of each dissected sample was homogenized in 1 mL of PBS using a tissue grinder (Tissue lyser‐48). Protein assays were performed on each individual homogenate. The deuterated internal standard mixture was diluted to 0.5 ng/μL in ethanol, and 10 μL (5 ng) was added. For metabolites, homogenates of ovarian tissue were extracted after addition of the internal standard mixture. The pH of the solution was adjusted to 3.0 using 1 mol/L NH4Ac, and liquid‐liquid extraction of the mixture was carried out twice using hexane:methyl t‐butyl ether (50:50, v/v). The upper layer was transferred to a new glass tube and evaporated under a gentle stream of nitrogen after centrifugation at 1500 *g* for 10 minutes at 4°C. The residue was dissolved in 50 μL of mobile phase A (water:acetonitrile:formic acid, 63:37:0.02, v/v/v) and stored at −80°C until analysis.

Samples were separated on a Phenomenex Kinetix C18 column (100 × 2.1 mm, 2.6 μm) at 350 μs/min using a gradient comprising mobile phase A and mobile phase B (acetonitrile:isopropanol, 50:50, v/v) as follows: The programme was initially set to 100% A, reached 92% A in 6 minutes and remained at 45% A from 6.5 to 10 minutes. The programme then switched to 100% B at 13 minutes and returned to 100% A from 14 to 14.5 minutes. MS analysis was performed on an AB Sciex 5500 QTrap hybrid quadrupole linear ion trap mass spectrometer in negative‐ion mode. Data acquisition and analysis were performed using Analyst software (AB SCIEX).

### 2.3 Cell culture

KGN cells were maintained in DMEM/F12 (Gibco, Grand Island, NY) supplemented with 10% FBS and 1% PS (penicillin/streptomycin) in 12‐well dishes at a density of 10^5^ cells/well for 24 hours at 37°C in 5% CO_2_. For AA (Sigma, Saint Louis, USA) and testosterone (ACROS ORGANICS, New Jersy, USA) stimulation, we added AA (100 ng/mL, 500 ng/mL and 1000 ng/ml according to the results in rat ovary) and testosterone (10 nmol/L, 50 nmol/L and 100 nmol/L) for 24 hours.

### 2.4 RNA isolation and qRT‐PCR

Total RNAs were isolated from ovarian tissues and KGN cells using TRIzol reagent (Invitrogen) and purified using the Qiagen RNeasy Mini Kit (Qiagen). Then, isolated RNAs were reverse‐transcribed into cDNA using a Superscript III First‐Strand Synthesis System (TaKaRa, Japan). Quantitative PCR (qPCR) was performed in triplicate using a LightCycler instrument (Roche Diagnostics) with SYBR Premix Ex Taq (TaKaRa). Gene expression primers used for the assays are indicated in Table S1. Sample cDNA expression levels were normalized to that of the reference gene GAPDH with the formula 2^−▵▵Ct^.

### 2.5 Western blot analysis

Briefly, ovarian tissues were homogenized in RIPA lysis buffer with 1% phenylmethylsulfonyl fluoride (PMSF). Proteins (50 μg) were separated on 10% SDS‐PAGE gels and then transferred to polyvinylidene fluoride (PVDF) membranes. After being blocked in 5% nonfat dried milk for 2 hours at room temperature, the membranes were blotted with rabbit anti‐COX2 (2169, 1:1000 dilution; Epitomics), anti‐cPLA2 (2832, 1:1000 dilution; Cell Signaling Technology, CST) or anti‐phosphorylated cytosolic phospholipase A2 (p‐cPLA2) (2831, 1:1000 dilution; CST) primary antibodies at 4°C overnight. The membranes were then incubated with the appropriate secondary antibody for 2 hours at room temperature. Protein bands were visualized via an enhanced chemiluminescence (ECL) kit (GE, USA) and quantified with AlphaEaseFC (Alpha Innotech).

### 2.6 Immunohistochemical analysis

Briefly, 4‐ìm‐thick frozen ovarian sections were prepared by conventional techniques. Primary anti‐COX2 (GB13077, 1:500 dilution; Guge Biotechnology, Wuhan, China) and anti‐cPLA2 (ab58375, 1:500 dilution; Abcam) antibodies were applied, and sections were incubated overnight at 4°C. After rinsing in PBS for 15 minutes, the appropriate secondary antibody was applied, and sections were incubated for 45 minutes at room temperature. The sections were then incubated with avidin‐biotinylated peroxidase complexes in PBS for 30 minutes. Reactions were revealed using diaminobenzidine tetrahydrochloride as the peroxidase substrate. Sections were rinsed in PBS after each immunostaining step. Finally, the sections were counterstained with haematoxylin, dehydrated and mounted in permanent mounting medium.

### 2.7 Steroid ELISA

The levels of oestrogen and progesterone in the spent media were measured using the Quantitative Diagnostic Kit (ELISA) (XITANG, Shanghai, China) according to the manufacturer's instructions.

### 2.8 Statistical methods

All data were analysed with SPSS software (version 18.0, SPSS, Chicago, IL), and measurement data are presented as the mean ± SEM. Data with non‐normal distributions were analysed after logarithmic transformation. Intergroup comparisons were performed using one‐way analysis of variance. *P* values < .05 were considered statistically significant.

## 3 RESULTS

### 3.1 Endocrinological and metabolic characteristics of the study subjects

No significant changes in fasting blood glucose levels were observed in the 3 groups, while blood glucose levels were significantly higher in the PCOS group than in the other 2 groups 30 minutes after glucose stimulation (*P* < .05; Figure S1B). As shown in Table S2, significantly higher fasting insulin and homeostasis model assessment—insulin resistance (HOMA‐IR) levels were found in the PCOS group compared to those in the CON and HF groups (*P *<* *.05). Compared to that of the controls, HF and PCOS rats had elevated total serum FFA levels (*P *<* *.05), and the level of the inflammatory indicator TNFα in the PCOS group was higher than that in the CON group (*P *<* *.05).

### 3.2 Alterations of PUFAs and their metabolites in PCOS rats

**Figure 1:**
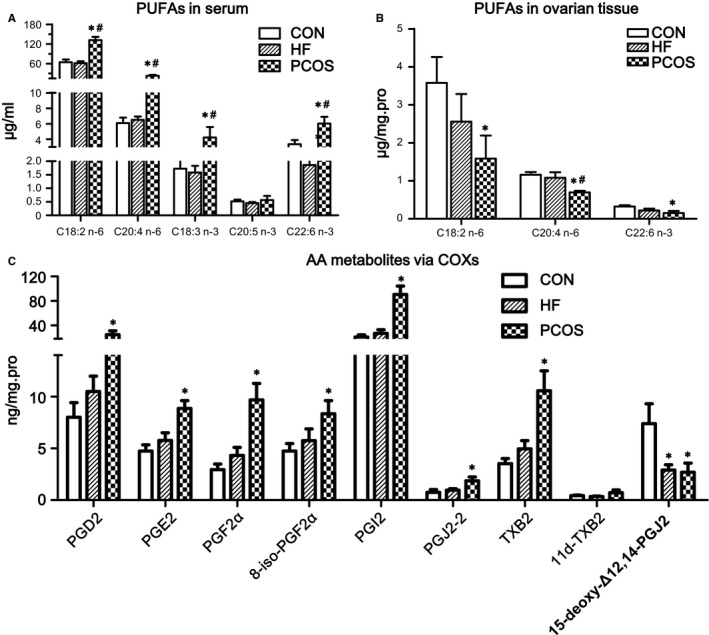
Alterations of PUFAs and their metabolites in PCOS rats. Comparisons of changes in serum (A) and ovarian tissue (B) PUFA levels among the 3 groups. (C) Changes in AA metabolites by COX in the 3 groups. *Compared with the CON group, *P *<* *.05; ^#^Compared with the HF group, *P *<* *.05. Serum PUFA levels are presented directly according to their concentrations. Because ovarian PUFA levels were measured in homogenized ovarian tissue, we adjusted the ovarian PUFA levels by total protein to make them comparable

As shown in Figure 1A, 5 kinds of PUFAs were detected and quantified in rat sera, LA (C18:2 n−6), AA (C20:4 n−6), α‐linolenic acid acid (ALA, C18:3 n−3), eicosapentaenoic acid (EPA, C20:5 n−3) and docosahexaenoic acid (DHA, C22:6 n−3). In addition to EPA, levels of the other 4 PUFAs were higher in the PCOS group than in the CON and HF groups, and these differences were statistically significant (*P *<* *.05). Changes in serum PUFA concentrations were not obvious between the CON and HF groups. Furthermore, only 3 kinds of PUFAs were detected in ovarian tissues, LA, AA and DHA, and their concentrations were lower than those in serum (*P *<* *.01). The ovarian levels of the 3 above‐mentioned PUFA types exhibited a descending tendency in the CON, HF and PCOS groups, respectively, and differences between the CON and PCOS groups were significant (*P *<* *.05). Moreover, the ovarian concentration of AA in the HF group was also significantly higher than that in the CON group (Figure 1B).

As shown in Figure 1C, compared with the CON group, the levels of AA metabolites, such as PGD2, PGE2, PGF2α, 8‐iso‐PGF2α, PGI2, PGJ2‐2, TXB2 and 11d‐TXB2, via the COX pathway in ovarian tissues of the PCOS group were significantly increased (*P *<* *.05) except for 15‐deoxy‐Δ12,14‐PGJ2. The ovarian tissue levels of AA metabolites by the lipoxygenase (LOX) and P450 (cytochrome P450, P450) pathways were not obviously different among the 3 groups (Figure S2).

### 3.3 Expression of key enzymes in AA metabolism

**Figure 2:**
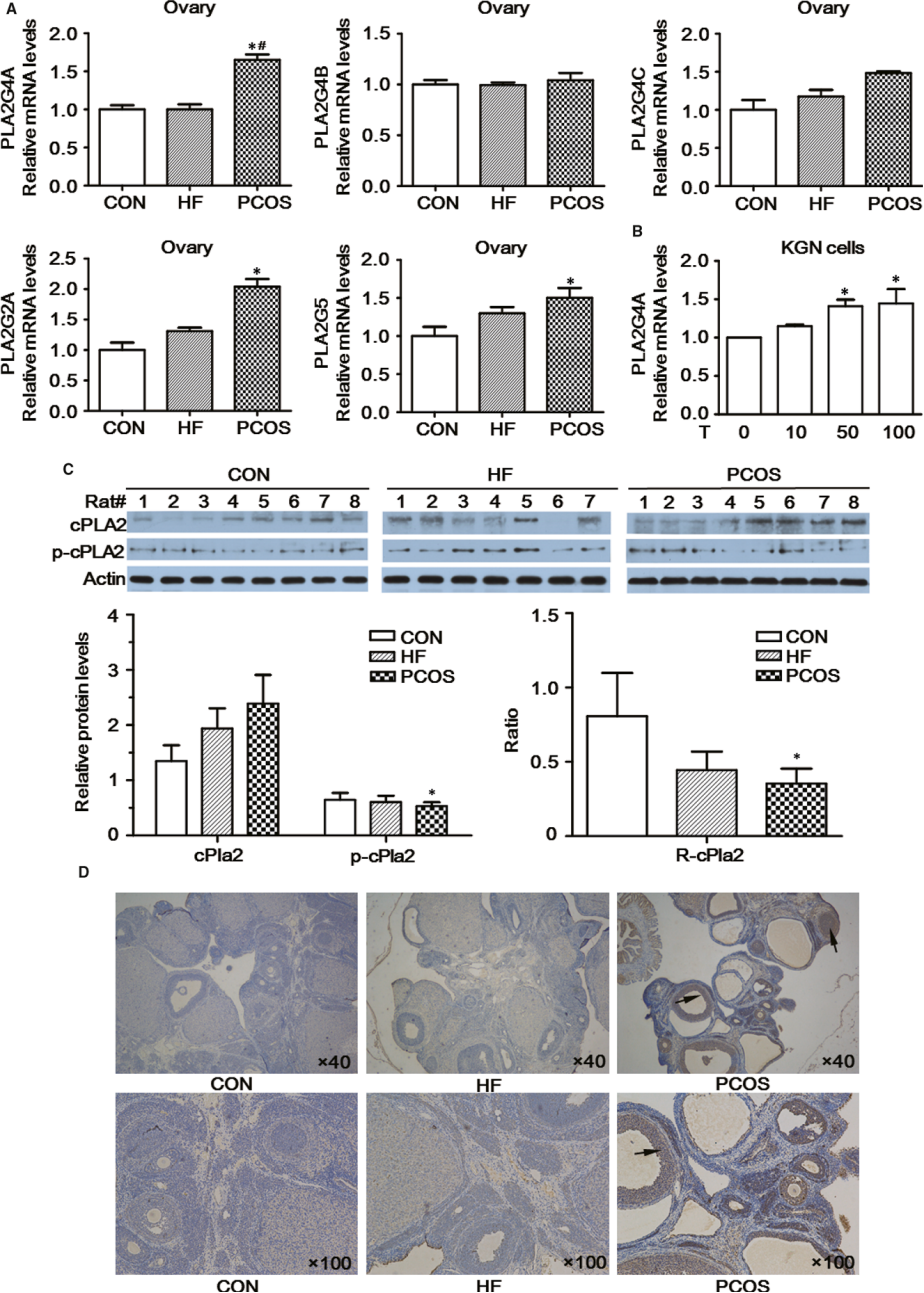
cPLA2 expression in rat ovarian tissue. A, mRNA expression levels of various PLA2 subtypes in rat ovarian tissue. B, mRNA expression levels of various PLA2G4A in KGN cells. C, Immunohistochemical evaluation of cPLA2 in rat ovarian tissue. D, cPLA2 and p‐cPLA2 protein contents in rat ovarian tissue as detected by Western blotting. R‐cPla2: ratio of p‐cPLA2/total cPLA2. Testosterone (10 nmol/L, 50 nmol/L and 100 nmol/L) was added to the KGN cells for 24 h. *Compared with the CON group or DMSO group, *P *<* *.05; ^#^Compared with the HF group, *P *<* *.05

As shown in Figure 2A, the mRNA levels of all the PLA2 subtypes were higher in ovarian tissues of the PCOS group than in those of the CON and HF groups. More specifically, significantly higher expression of PLA2G4A was found in the PCOS group compared with that in the CON and HF groups (*P *<* *.05). The mRNA levels of PLA2G4A from KGN cells were also increased after the stimulation with testosterone (Figure 2B). The gene expression levels of PLA2G2A and PLA2G5 in the PCOS group were also higher than those in the controls (*P *<* *.05). Second, the protein expression levels of cPLA2 in rat ovaries exhibited a gradually increasing trend in the CON, HF and PCOS groups, respectively, while the p‐cPLA2 protein expression levels and the p‐cPLA2/cPLA2 ratio (R‐cPLA2) gradually decreased. Both the p‐cPLA2 protein expression levels and R‐cPLA2 were significantly lower in the PCOS group than those in the CON group (*P *<* *.05; Figure 2C). Immunohistochemistry analysis revealed results similar to those of Western blot (Figure 2D).

**Figure 3:**
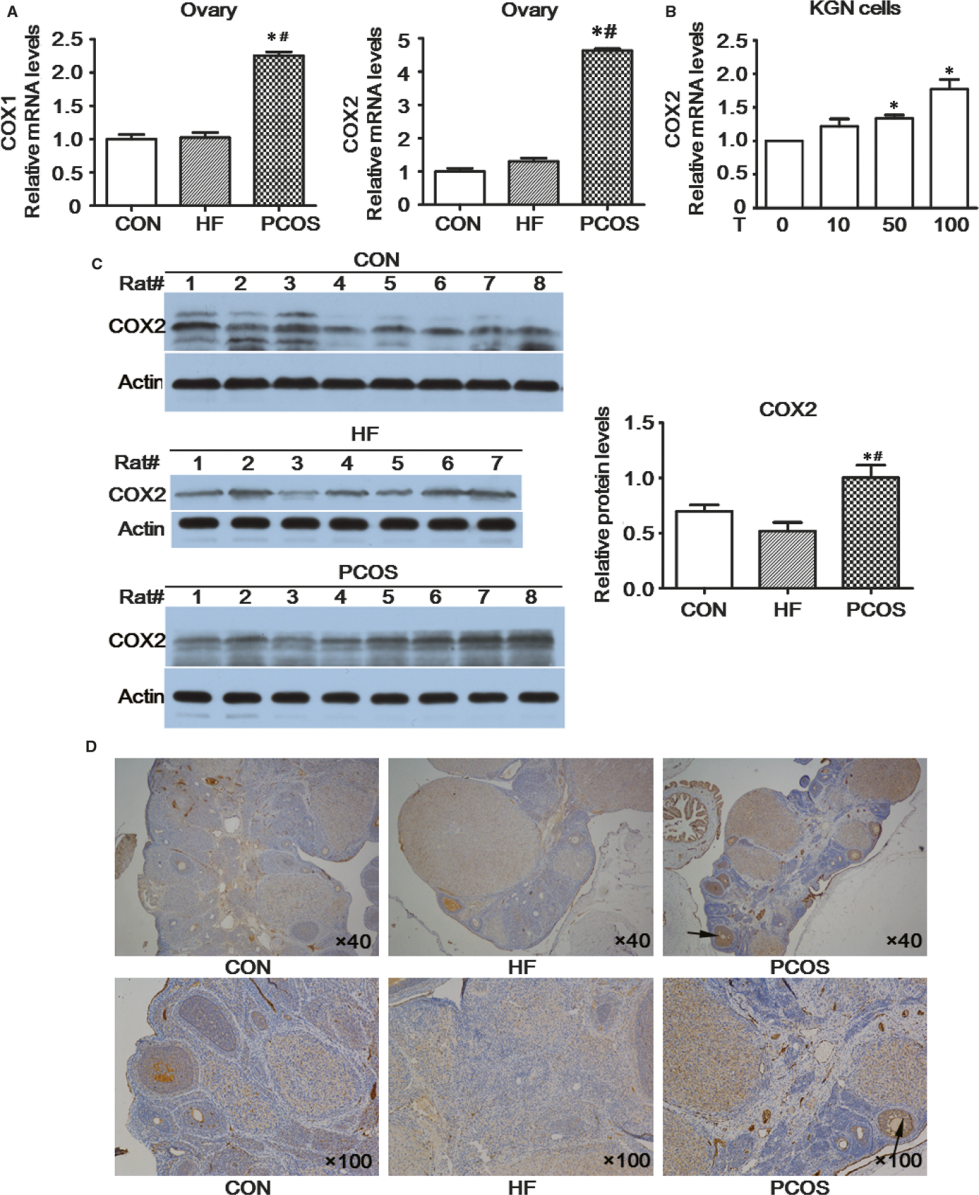
COX expression in rat ovarian tissue. A, COX gene expression levels in rat ovarian tissue. B, mRNA expression levels of various COX2 in KGN cells. C, Immunohistochemical evaluation of COX2 in rat ovarian tissue. D, COX2 protein content in rat ovarian tissues as determined by Western blot. Testosterone (10 nmol/L, 50 nmol/L and 100 nmol/L) was added to the KGN cells for 24 h. *Compared with the CON group or DMSO group, *P *<* *.05; ^#^Compared with the HF group, *P *<* *.05

COX1 and COX2 mRNA expression levels in ovarian tissues of the PCOS group were higher than those of the CON and HF groups (*P *<* *.05; Figure 3A). Western blot indicated that COX2 protein expression levels in PCOS rat ovaries were significantly higher than those in the other 2 groups (*P *<* *.05) and were not different between the CON and HF groups (Figure 3C). The mRNA levels of COX2 from KGN cells were higher after the stimulation with testosterone (Figure 3B). As shown in Figure 3D, darkened brown granules were located in mainly granulosa cells of antral follicles and corpus lutea. Several antral follicles were observed in ovarian tissues of the PCOS group, with staining of antral follicle darkening significantly, and no obvious changes in staining of the CON and HF groups were observed.

### 3.4 Estradiol and progesterone levels after testosterone and AA stimulation

**Figure 4:**
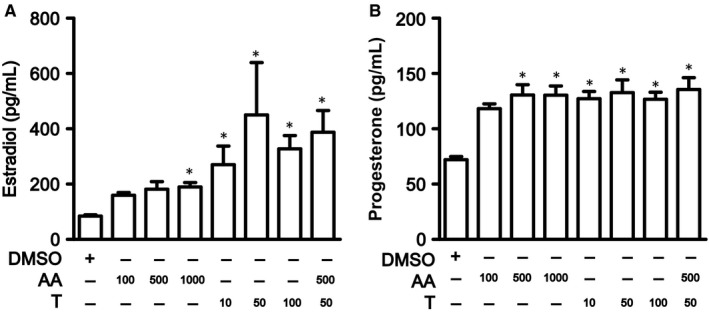
Estradiol and progesterone level of KGN cells after testosterone and/or AA stimulation. Estradiol (A) and progesterone (B) produced by KGN cells after testosterone and/or AA stimulation. AA (100 ng/mL, 500 ng/mL and 1000 ng/mL) and testosterone (10 nmol/L, 50 nmol/L and 100 nmol/L) were added to the KGN cells for 24 h. *Compared with the DMSO group, *P *<* *.05

We then stimulated KGN cells with testosterone and/or AA. The estradiol level was increased after either testosterone or AA stimulation, as well as in combination group (Figure 4A). Moreover, the progesterone level was increased after testosterone and/or AA stimulation (Figure 4B).

## 4 DISCUSSION

According to a previous study,^17^ we established a PCOS rat model by treating rats fed a high‐fat diet with andronate, and they then showed ovarian and metabolic features of PCOS. We found higher serum AA levels but lower ovarian tissue AA levels in PCOS rats compared with those of CON and HF rats. In addition to 15‐deoxy‐Δ12,14‐PGJ2, levels of AA metabolites via the COX pathway were increased in PCOS rats. Further analysis of the mechanism underlying this phenomenon showed a correlation between decreased p‐cPLA2 expression and increased COX2 expression in the ovarian tissues of PCOS rats, which might have led to alterations in the levels of AA and its metabolites (PGs).

Polycystic ovary syndrome rats exhibited higher levels of total FFAs in serum compared to those of the controls, which was consistent with results obtained from PCOS patients undergoing in vitro fertilization.^4^ We next quantified 4 kinds of PUFAs in circulation by lipidomics, and PCOS rats exhibited higher levels of all 4 types than control and HF rats. Nevertheless, only 3 kinds of PUFAs were detected in ovarian tissues by the same method, and descending trends in the concentrations of LA, AA and DHA were observed in the control, HF and PCOS rats, respectively, with variation in AA levels being the most significant. In our research, a series of PGs were also significantly elevated in PCOS rats, while differences in AA metabolites via the LOX and P450 pathways among the 3 groups were not observed.

N−6 fatty acids are widely recognized as pro‐inflammatory agents,^21^, ^22^ and AA (C20:4 n−6) has pronounced effects on gene expression, leading to changes in metabolism, cell growth and differentiation. Phospholipase A2 (PLA2) selectively catalyses hydrolysis of the sn‐2‐ester bond in membrane phospholipids, which can be stimulated by signals from a vast range of inflammatory markers, cytokines, growth factors and hormones and results in AA formation. The conversion of AA is further processed by downstream metabolic enzymes, such as COXs (COX1 and COX2), P450 and LOX, into eicosanoids, such as PGs and leukotrienes, which are terminal products that play roles in a variety of biological activities via their respective synthetases.^23^ Hence, we focused on PLA2/AA/COX pathways, which are the most relevant to ovarian function.

Many forms of PLA2 have been identified to date,^24^ but only 3 are capable of cleaving AA from membrane phospholipids: secretory PLA2 groups (PLA2G2A, PLA2G5) and cytosolic PLA2 (cPLA2, PLA2G4).^25^ cPLA2 (PLA2G4) is specific for cleavage of AA from the second position of membrane phospholipids, whereas PLA2G2A and PLA2G5 hydrolyse any fatty acid at the second position. In our recent study, mRNA expression levels of PLA2G2A, PLA2G5 and PLA2G4A were higher in the ovaries of PCOS rats than in the controls, with a significant increase in PLA2G4A expression being observed in the HF group. The protein expression levels of cPLA were also elevated, as determined by Western blot and immunohistochemical analyses, but the p‐cPLA2 protein expression level and R‐cPLA2 were significantly decreased in the ovaries of PCOS rats compared to those of the controls. cPLA2 must be phosphorylated to cleave AA from membrane phospholipids, and elevated intracellular calcium levels stimulate cPLA2 activity independent of the phosphorylation status,^26^ indicating the decreased production of AA via cPLA2 catalysis in the ovarian tissues of PCOS rats.

Notably, PGs have important impacts on both the development of reproductive events and chronic inflammation, which are hallmarks of PCOS. The key regulatory step in PG biosynthesis is enzymic conversion of the fatty acid precursor by COX enzymes (COX1 and COX2). COX1 is constitutively expressed in theca cells but absent in granulosa cells, whereas COX2 is inducible by luteinizing hormone (LH)/human chorionic gonadotropin (hCG) only in granulosa cells.^27^ COX2 expression levels in ovaries of PCOS rats were elevated at both the gene and protein level in our research, and a synchronous increase in the levels of several PGs was also observed. This result contrasts with those of others reporting that the COX2 expression level and PGE concentration were not altered, but PGF2α levels were elevated in follicular fluid and granulosa cells in patients with PCOS after superovulation.^28^ This difference might be related to differences in the study objects and interventions.

Amalfi et al 29 reported that a higher dose of testosterone induced a pro‐inflammatory state in ovarian tissue mediated by increased levels of PGE and protein expression of COX2, the limiting enzyme of PG synthesis. Injection of chorionic gonadotropin into female rats enhanced the mRNA expression of COX2, leading to increased serum estradiol levels, and such effects were further amplified after hyperandrogenism.^30^ Serum testosterone and LH concentrations were elevated in PCOS rats,^17^ consistent with our current results. We also demonstrated that COX2 was expressed in rat granulosa cells, as rats fed a high‐fat diet that was exposed to hyperandrogenism exhibited increased ovarian COX expression in a manner consistent with a role of these enzymes in PG production. Because primate follicular fluid PG levels increase 100‐fold in response to ovulatory gonadotropin surges,^31^, ^32^ AA must be rapidly mobilized for periovulatory PG production.^33^ PLA2G4A and COX2 mRNA expressions were also observed to be coinduced in the proresolving phase of the acute inflammatory process.^34^ A cPLA2‐selective inhibitor decreased both PLA2 activity in monkey granulosa cell lysates and PGE2 accumulation in human granulosa‐lutein cell cultures.^35^ However, no changes in mRNA abundance corresponding to COX2, similar PGE levels and diminished PGF2α levels, were observed in PCOS follicles after hCG exposure. Considering the results obtained herein, decreased expression of the upstream bioactive p‐cPLA2 and increased activity of the downstream COX enzyme in ovarian tissues may have accounted for the decreased levels of AA found in the PCOS group.

Notably, some limitations of this study do exist. Due to the heterogeneity of PCOS, creating a single‐animal model that expresses all PCOS characteristics is difficult.^36^ Second, although we have elucidated changes in key genes involved in alterations of AA and its metabolites in ovarian tissues of PCOS rats, additional studies are needed to explore the exact effects and mechanisms of AA and its metabolites underlying oocyte developmental competence during ovulation.

In conclusion, we demonstrated that ovarian levels of PUFAs in PCOS rats were decreased, with changes in AA being the most significant, although serum PUFA levels were increased accordingly. Correlation between decreased p‐cPLA2 activity and increased COX expression might result in lower AA concentrations in ovarian tissues of PCOS rats. However, the exact effects and mechanisms of AA and its metabolites underlying ovarian function remain unelucidated.

## CONFLICT OF INTEREST STATEMENT

The authors confirm that there are no conflict of interests.

## AUTHORS’ CONTRIBUTIONS

Rong Huang and Xinli Xue performed the research; Shengxian Li, Yuying Wang and Yun Sun analysed the data; Wei Liu, Huiyong Yin and Tao Tao designed the research study; and all authors participated in writing the manuscript.
