# Supplementary material for: Alterations of polyunsaturated fatty acid metabolism in ovarian tissues of polycystic ovary syndrome rats
Source: J Cell Mol Med. 2018 Mar 30;22(7):3388–96. doi: 10.1111/jcmm.13614 (PMC6010729; doi:10.1111/jcmm.13614)
Supplement: Supplementary file 1 [file JCMM-22-3388-s001.docx]

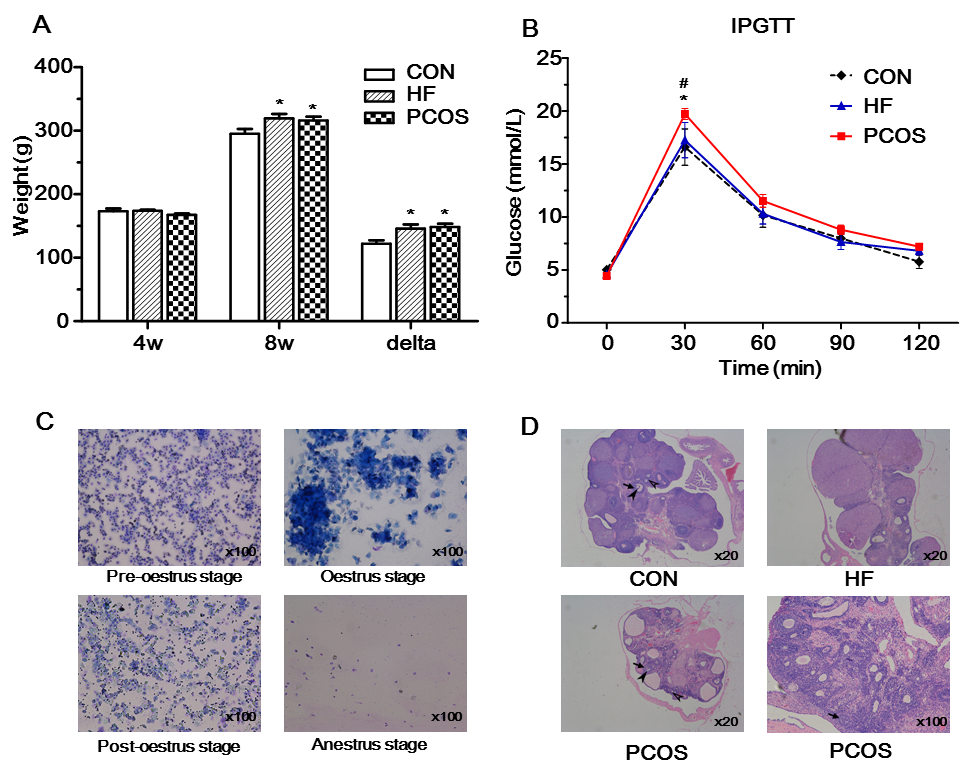


**Supplementary Figure 1.** Characteristics of the PCOS rat model. Body weight changes (A) and blood glucose concentrations during IPGTT (B) in the three groups. Body weights of rats from all 3 groups were not different before establishment of the model. Compared with the controls, rats of the HF and PCOS groups exhibited significantly increased mean body weights at the conclusion of the study (P=0.009, P=0.004, respectively). (C) Reproductive cycles of rats as determined by vaginal smears. Rats of the control group exhibited regular estrous cycles with pre-estrus, estrus, post-estrus and anestrus occurring in the proper order, whereas rats of the two experimental groups had prolonged or irregular estrous cycles, most of which showed a constant diestrus smear. (D) Ovarian morphology of rats. Normal ovarian structures with follicles and corpora lutea in various stages were observed in the control group, and no follicular cysts were observed. The microscopic manifestation of ovaries in the HF group was similar to that of the CON group, as occasional small vesicular follicles were observed. Rats of the PCOS group demonstrated significantly increased primary and secondary follicles, increased and poorly organized granulosa cell layers, theca cell proliferation and mesenchymal thickening. These rats also exhibited significantly reduced corpus lutea in ovarian tissues, multiple growing follicular cysts, and decreased granulosa cell layers in vesicular follicles.

IPGTT: intra-peritoneal glucose tolerance test.

Primary follicles: ; secondary follicles: ; granulosa cell: .


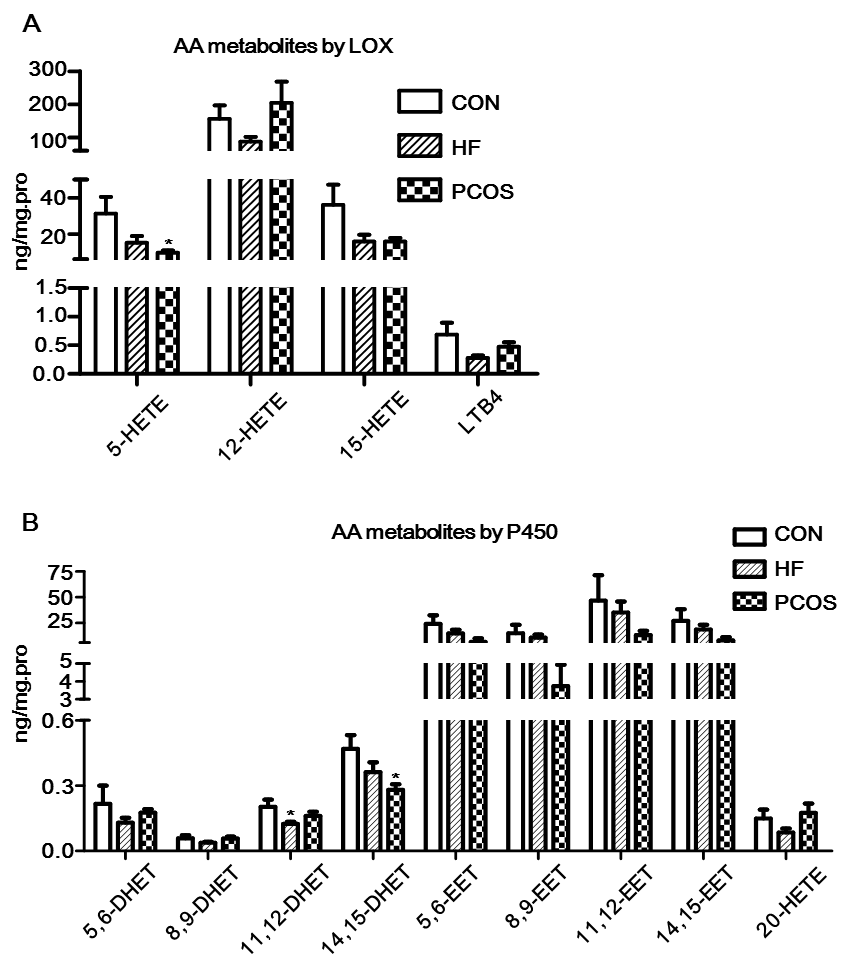


**Supplementary Figure 2.** Comparisons of AA metabolites by LOX and P450. AA metabolites by LOX (A) and by P450 (B). *: Compared with the CON group, P < 0.05.
