# Supplementary material for: Alterations of polyunsaturated fatty acid metabolism in ovarian tissues of polycystic ovary syndrome rats
Source: J Cell Mol Med. 2018 Mar 30;22(7):3388–96. doi: 10.1111/jcmm.13614 (PMC6010729; doi:10.1111/jcmm.13614)
Supplement: Supplementary file 2 [file JCMM-22-3388-s002.docx]

**Supplementary Table 1. Sequences of oligonucleotide primers used for Real-Time PCR.**

| **Gene** | **Forward (5'-3')** | **Reverse (5'-3')** |
| --- | --- | --- |
| **PLA2G2A** | CCTTTGGCTCAATTCAGGTCC | CACAATGGCAACCGTAGAAGC |
| **PLA2G5** | GATGCACGACCGTTGTTATGG | AGCACAAAGCCTCACTGGACAG |
| **PLA2G4A** | ATGCTAATGGCCTTGGTGAGTG | GCGTCGAGTTCATCATCATCG |
| **PLA2G4B** | GAGCGCAAAGTTCAACTTGTGG | GCTGGATAATGGAAGCGGAAAG |
| **PLA2G4C** | TACTTTGCCACTGGACTCCAGG | ATAGCACTCAGCACCCCAAGAC |
| **COX1** | TGCCCTCTGTACCCAAAGACTG | AGCAAACAAGACGTTGGTCCC |
| **COX2** | CACGGACTTGCTCACTTTGTTG | AGCGTTTGCGGTACTCATTGA |
| **GAPDH** | TCCTGCACCACCAACTGCTTAG | AGTGGCAGTGATGGCATGGACT |

PLA2: phospholipase A2, COX: cyclooxygenase.

**Supplementary Table 2. Endocrinological and metabolic characteristics of the rats.**

| **Variables** | **CON** |  | **HF** |  | **PCOS** |  |
| --- | --- | --- | --- | --- | --- | --- |
| **TT (nmol/L)** | 0.81±0.54 | | 0.73±0.47 | | 34.99±9.34^*#^ | |
| **DHEAS (μg/mL)** | 0.07±0.02 | | 0.06±0.03 | | 0.04±0.02^*^ | |
| **E2 (pg/ml)** | 157.79±54.27 | | 200.39±62.61 | | 125.39±29.07^#^ | |
| **FINS (μIU/ml)** | 5.51±3.31 | | 11.37±3.78^*^ | | 16.65±4.58^*#^ | |
| **HOMA-IR** | 1.23±0.73 | | 2.27±0.72^*^ | | 3.27±0.90^*#^ | |
| **TNFα (pg/ml)** | 9.97±4.65 | | 14.31±3.05 | | 25.08±7.99^*^ | |

^*^: Compared with CON group, P < 0.05; ^#^: Compared with HF group, P < 0.05.

TT: total testosterone; DHEAS: sulfated dehydroepiandrosterone; E2: estradiol; FINS: fasting insulin; HOMA-IR: homeostasis model assessment-insulin resistance; TNFα: tumor necrosis factor α.
